# Optical Coherence Tomography Angiography Flow Signal in Non-Treatment-Naïve Patients with Neovascular Age-Related Macular Degeneration Treated with Faricimab

**DOI:** 10.3390/medicina61020260

**Published:** 2025-02-03

**Authors:** Max Brinkmann, Tom Müller, Marco Köster, Jakob Schweighofer, Mathis Danckwardt, Giuseppe Giannaccare, Paola Marolo, Enrico Borrelli, Michele Reibaldi, Yosuf El-Shabrawi, Mario Damiano Toro

**Affiliations:** 1 Department of Ophthalmology, Klinikum Klagenfurt, 9020 Klagenfurt, Austria; tom.mueller@kabeg.at (T.M.); yosuf.el-shabrawi@kabeg.at (Y.E.-S.); 2 Department of Ophthalmology, Universitätsklinikum Schleswig-Holstein, 23564 Lübeck, Germany; mathis-danckwardt@web.de; 3 Faculty of Medicine, Medical University of Graz, 8010 Graz, Austria; marco.koester@gmx.at; 4 Department of Ophthalmology, Universitätsklinikum Wiener Neustadt, 1090 Vienna, Austria; jakob.schweighofer@meduniwien.ac.at; 5 Eye Clinic, Department of Surgical Sciences, University of Cagliari, 09124 Cagliari, Italy; giuseppe.giannaccare@unica.it; 6 Department of Ophthalmology, University of Turin, 10126 Turin, Italy; paola@marolo.com (P.M.); borrelli.enrico@yahoo.com (E.B.); michele.reibaldi@unito.it (M.R.); 7 Eye Clinic, Public Health Department, University of Naples Federico II, 80133 Naples, Italy

**Keywords:** Faricimab, choroid, OCTA, anti-VEGF, angiopoietin 2, AMD

## Abstract

*Background and Objectives:* Age-related macular degeneration (AMD) remains a leading cause of legal blindness. Anti-Vascular Endothelial Growth Factor (VEGF) agents are the first-line treatment for neovascular AMD (nAMD). The choroid plays a key role in AMD and is affected by the anti-VEGF treatment. Faricimab, a bispecific antibody additionally targeting angiopoietin 2 (Ang2), was recently approved for nAMD treatment. This study investigates the effect of Faricimab on choroidal flow signal. *Materials and Methods:* Optical coherence tomography angiography images of 29 nAMD eyes were examined retrospectively. Patients had received treatment with other anti-VEGF agents before Faricimab application. The flow signal in the choroid was measured before, after one and after a series of ≥2 Faricimab injections. *Results:* The flow signal decreased significantly (*p* = 0.026) at the choriocapillaris (CC) level after ≥2 injections. The flow signal did not show a significant change in Haller’s layer but increased slightly in Sattler’s layer (*p* = 0.034). *Conclusions:* In conclusion, our results show that the flow signal, especially at the CC level, changed during treatment. Despite the known influence of anti-VEGF treatment on the choroid, auxiliary inhibition of Ang2 might enhance this effect. Due to the retrospective nature, moderate sample size and non-treatment, naïve patients, care must be taken while interpreting our observations. Prospective studies with larger sample sizes and treatment-naïve patients will be needed.

## 1. Introduction

Age-related macular degeneration (AMD) is a leading cause of blindness, affecting more than six million people globally [1,2]. It is a disease that affects people older than 55 years by definition. Rapid population growth and demographic changes facilitated by progress in the fields of medicine, public health and social welfare have led to a significant increase in the number of individuals aged over 60 years [3]. Age-related macular degeneration continues to pose a significant public health challenge in spite of advancements in treatment and diagnostics. The psychosocial impact of vision loss caused by age-related macular degeneration is evident, with individuals ranking it among the most undesirable health outcome [4]. Visually demanding tasks like reading, driving and facial recognition are dependent on intact central vision. As advanced AMD can cause significant central vision loss, it is linked to a diminished quality of life, reduced mobility and independence and heightened rates of depression and falls [5].

The precise pathogenesis of AMD remains elusive, yet it is likely the result of intricate and multifaceted processes [6]. Cumulative oxidative damage plays a pivotal role in instigating anatomical and physiological alterations within the photoreceptors, the RPE, Bruch’s membrane and the choriocapillaris as an integral part of the natural aging progression. Over the initial decades of life, RPE cells gradually accumulate intracellular debris [7]. The primary source is the accumulation of undegradable byproducts stemming from the visual cycle. The buildup is believed to induce cellular injury, disrupt RPE function and result in irregularities in the deposition of the extracellular matrix [8].

Drusen are deposits of extracellular, lipid-rich membranous debris that form between the basal lamina of the RPE and the inner collagenous layer of Bruch’s membrane as dysfunction in the RPE intensifies. They represent a hallmark lesion of age-related macular degeneration. Drusen material may also accumulate in a thin layer termed basal linear deposits [9]. Components within drusen are thought to initiate the activation of the complement system, a fundamental aspect in the pathogenesis of drusen formation and the subsequent progression of pathological changes in AMD. As a result, it is hypothesized to induce a loss of endothelial cells in the choriocapillaris, leading to subsequent choriocapillaris degeneration [10]. This loss in the choriocapillaris is believed to facilitate oxidative injury to the overlying RPE and outer retinal structures, fostering inflammation and contributing to the advancement of AMD.

Ultimately, this cascade of pathophysiological events results in the demise of photoreceptor cells, as the cumulative burden of oxidative injury resulting from hypoxia and chronic inflammation surpasses the capacity of the RPE. The intricate interplay of extracellular debris deposition, oxidative damage, cellular dysfunction, complement system activation and choriocapillaris degeneration collectively delineates the complex landscape of AMD pathogenesis. Further unraveling these processes holds the key to advancing our understanding of AMD and developing targeted therapeutic strategies to intervene in its progression [11].

To enhance our understanding of AMD pathophysiology and especially for monitoring the applied treatment, optical coherence tomography (OCT) and optical coherence tomography angiography (OCTA) have become major diagnostic tools. This non-invasive technology enables us to create high-resolution images of the retina and the retinal and choroidal blood flow. Thus, these technologies deliver important insights not only into AMD but also systemic diseases [12].

As mentioned earlier, there is a strong indication that impaired choroidal flow signal plays a significant role in AMD pathophysiology. Several histopathologic studies have described choroidal dysregulation even in early and intermediate AMD promoting angiogenesis and vascular remodeling [13,14,15,16]. Increased choroidal and especially choriocapillaris (CC) flow impairment appears to play a major part and show a strong association with AMD eyes in comparison to non-AMD eyes [17]. There is evidence, that in the setting of neovascular AMD (nAMD), spatial distribution impairment of the CC is linked to growth of abnormal blood vessels in the sub-retinal pigment epithelium (RPE) cell spaces, thereby forming macular neovascularization (MNV) [15].

Vascular endothelial growth factor (VEGF) secreted by the RPE plays another key role in the formation of MNV. VEGF is the major protein to target in multiple diseases involving MNV [18,19]. Results about the impact of anti-VEGF drugs on the choroid are controversial. Some studies found that the long-term administration of anti-VEGF agents could promote decreased vascular density especially in the CC [20,21]. Furthermore, using OCT and OCT angiography (OCTA), previous studies have proven that intravitreally applied Anti-VEGF agents have an impact on the choroid, including CC flow signal [22,23,24].

Faricimab has been approved by the food and drug administration (FDA) for the treatment of exudative AMD and diabetic macular edema (DME) in 2022 [25]. It is the first FDA-approved intraocular drug to target angiopoetin2 (Ang2) in addition to VEGF. Ang2 is part of the Ang/Tie pathway and plays a significant role in the regulation of vascular homeostasis, the modulation of vascular permeability and in neoangiogenic and proinflammatory processes [26,27]. Its influence on choroidal flow signal has not yet been evaluated in vivo. This is especially interesting because of Ang2 involvement in vascular homeostasis. Therefore, this study aims to investigate choroidal flow signal before and after the intravitreal application of Faricimab using OCTA.

## 2. Materials and Methods

This retrospective study analyzed 29 eyes of 25 subjects receiving intravitreal Faricimab injections for the treatment of nAMD at the Department of Ophthalmology, Klinikum Klagenfurt, Austria, between November 2022 and October 2023. All patients had been treated with other anti-VEGF agents before the application of Faricimab. This study followed the ethical guidelines in line with the Declaration of Helsinki and was approved by the local review board.

All patients received a complete ophthalmologic examination, which included the measurement of best-corrected visual acuity (BCVA), intraocular pressure (IOP) and dilated ophthalmoscopy. Only patients with an IOP within the normal range (10–21 mmHg) were included. The exclusion criteria included (i) the presence of significant cataract; (ii) myopia greater than 3.00 diopters; (iii) a history of myocardial infarction or cerebrovascular disease within the last 6 months; (iv) an infection or inflammation of both eyes; (v) the presence of other comorbid retinal and/or macular diseases (e.g., diabetic retinopathy and retinal venous occlusion); (vi) and any optic neuropathy, including glaucoma.

Imaging was performed before (T1), after one (T2) and after a series of at least two (T3) Faricimab injections.

High-resolution spectral domain OCT images (Spectralis OCT, Heidelberg Engineering, Heidelberg, Germany) obtained before and during Faricimab treatment were analyzed. The Heidelberg Spectralis SD-OCT operates with a center wavelength of 850 nm (bandwidth 60 nm) and an A-scan rate of 85 kHz. For each OCT scan, a pattern of B-scans (*n* = 19), horizontally oriented and centered over the fovea in a 20° × 15° (5.7 × 4.2 mm) area, were acquired. CRT was determined using the macula thickness map tool in the Heidelberg software (Heidelberg Eye Explorer, Heidelberg Engineering, Heidelberg, Germany).

The OCTA acquisition was performed before and after Faricimab application using the ZEISS PLEX Elite 9000, swept-source OCT Angiography (Carl Zeiss AC, Jena, Germany). This device operates with a wavelength between 1040 and 1070 nm. It uses two different A-scan speeds between 100,000 and 200,000 A-scans/second. The device’s follow-up mode was used to assure measurements at the same location for both time points. Each imaging session included OCTA volumetric scans (6 × 6 mm^2^) of the posterior pole. OCTA scans with a strength index less than eight out of ten with significant motion artifact or shadowing effect were excluded from the analysis [28]. Due to the structure of our clinic, all medical retina patients are routinely assessed at the same time of day (between 08:00 and 12:00 a.m.). Therefore, potential biases due to physiological diurnal changes of the ocular flow signal were compensated for [29,30]. OCTA images were automatically segmented in all B-scans according to the manufacturer’s default setting. Automated segmentation was used to get 20 µm slabs of the choriocapillaris (CC). Manual segmentation (M.B.) was applied to Haller’s layer (HL) and Sattler’s layer (SL) according to previously published protocols [28,30]. The RPE layer was chosen as the reference layer. The SL-slab was defined as the space between 60 and 80 µm below the RPE, and the HL-slab was defined as the space between 120 and 140 µm below the RPE. Each acquired en face image was exported into ImageJ (NIH, Version 1.52e, Bethesda, Rockville, MD, USA) and binarized using the Otsu method, an automatic threshold selection from gray-level histograms in order to determine the percentage of white and black pixels [31]. As published before, the CC flow signal was calculated by recording the percentage of white pixels, while for HL and SL, the flow signal black pixels were considered [28,29,30].

Continuous factors are expressed as means ± standard deviations (SD), while categorical factors are expressed as percentages and number of occurrences. In order to assess changes in the observed parameters (BCVA, CRT, CC-flow, HL-flow and SL-flow) over time, multilevel random intercept regression models (mixed models) were created. These models account for the inter-eye correlation present in the data and consider the “repeated measure” data format. The observation times were specified as the two random levels of the models. For this method, our data is considered a small dataset. The model estimation was therefore carried out using the Restricted Maximum Likelihood algorithm with the approximate finite-sample distribution for Wald statistics for regression coefficients. This analysis was provided using the “reml” and “dfmethod (kroger)” options with the “mixed” command. Data were analyzed by Stata version 18.0 (StataCorp LP, College Station, TX, USA). Statistical significance was accepted at *p* < 0.05.

## 3. Results

A total of 29 eyes of 25 Caucasian patients receiving a series of at least two Faricimab injections (mean 3.3 ± 1.6) for the treatment of nAMD were included in the study. Eighteen patients were female, and seven patients were male. The cohort consisted of 12 right and 17 left eyes. The mean age was 78.4 ± 6.6 years (range 64–89 years, ±SD).

All patients had been treated with other anti-VEGF agents (Ranibizumab, Aflibercept and Brolucizumab) before changing to Faricimab due to poor treatment response (Table 1).

A minimum of 6 weeks passed between the application of the last drug and the first Faricimab injection. On average, patients had received 15.9 ± 15.3 intravitreal injections with anti-VEGF drugs before the first application of Faricimab (range 2–53, ±SD; Table 1). During the former treatment regimen, the latest time interval between injections was 5.4 ± 1.1 weeks (range 4–8, ±SD; Table 1).

Mean BCVA was 0.50 ± 0.3 (range 0–1.0 LogMAR, ±SD) at T1, 0.46 ± 0.3 (range 0–1.0 LogMAR, ±SD) at T2 and 0.50 ± 0.4 (range 0–1.0 LogMAR, ±SD) at T3. No significant change of best-corrected visual acuity (BCVA) was found between T1, T2 and T3.

Four eyes had received at least 2 Faricimab injections at T3, the rest (25 eyes) received at least 3 injections (Table 2).

**Figure 1:**
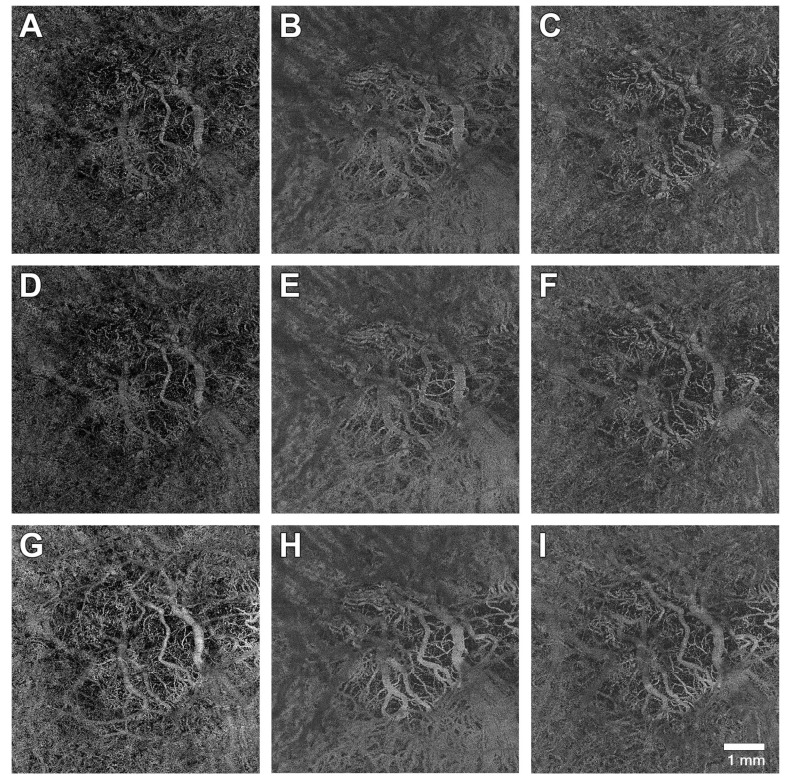
Optical coherence tomography angiography en face images at different levels taken at three different time points. (**A**–**C**) images were acquired prior to first Faricimab injection (T1). (**D**–**F**) images were taken four weeks after the first injection (T2). (**G**–**I**) images were shot after a series of at least two Faricimab injections (T3). (**A**,**D**,**G**) show the detected flow signal from an en face perspective at the level of the choriocapillaris. (**B**,**E**,**H**) are focused on Haller’s layer, and (**C**,**F**,**I**) represent Sattler’s layer.

Before the first Faricimab injection (T1), the mean flow signal of the choriocapillaris (CC) was 54.4 ± 4.0 (range 41.8–61.2%, ±SD, Table 3). After the first injection (T2), the CC flow signal was 52.9 ± 5.9 (range 32.6–61.7%, ±SD, Table 3). After a series of at least 2 Faricimab injections (T3), the CC flow signal was 51.97 ± 6.9 (range 33.7–59.1%, ±SD, Table 3). The flow signal of the CC decreased significantly between T1 and T3 (*p* = 0.05, Figure 1 and Table 3 and Table 4).

Before the first Faricimab injection (T1), the mean flow signal of Haller’s layer (HL) was 54.1 ± 4.8 (range 44.1–63.2%, ±SD, Table 3). After the first injection (T2), the HL flow signal was 55.2 ± 4.8 (range 48.1–63.3%, ±SD, Table 3). After a series of at least 2 Faricimab injections (T3), the HL flow signal was 55.8 ± 5.1 (range 45.4–69.6%, ±SD, Table 3). No significant change was found on the level of HL.

Before the first Faricimab injection (T1), the mean flow signal of Sattler’s layer (SL) was 54.7 ± 3.5 (range 49.2–64.7%, ±SD, Table 3). After the first injection (T2), the SL flow signal was 55.0 ± 4.8 (range 41.0–63.9%, ±SD, Table 3). After a series of at least 2 Faricimab injections (T3), the SL flow signal was 55.9 ± 4.4 (range 50.1–68.5%, ±SD, Table 3). No significant change of flow signal was found.

Before the first Faricimab injection (T1), the mean central retinal thickness (CRT) was 352 ± 82 (range 237–541 µm, ±SD, Table 3). After the first injection (T2), CRT was 292 ± 54 (range 206–433 µm, ±SD, Table 3). After a series of at least 2 Faricimab injections (T3), CRT was 301 ± 78 (range 204–507 µm, ±SD, Table 3). CRT decreased significantly between T1 and T2 (*p* < 0.001), as well as between T1 and T3 (*p* = 0.012, Table 3 and Table 4).

## 4. Discussion

This study sought to evaluate the influence of intravitreally administered Faricimab on choroidal flow signal using OCTA. Previous studies have highlighted the importance of the choroid and especially the choriocapillaris in the setting of AMD [13,16,32,33]. It is hypothesized that impairment of the CC is a key factor in the development and progression of AMD [16,33,34]. Furthermore, there is evidence that anti-VEGF drugs can cause structural and functional changes in the choroid [20,22,35]. Given that Faricimab is the first approved drug that not only targets VEGF but also Ang2, investigating its effect on the choroid is of interest.

Our results show that the CC flow signal significantly decreased after a series of at least two Faricimab injections, whereas the flow signal of HL and SL stayed unchanged.

Multiple studies found evidence for CC impairment in the setting of AMD. Histologic evidence suggests that CC degeneration impacts RPE viability [33]. Using light and electron microscopy, Biesemeier et al. described that CC breakdown occurs during normal aging but increases significantly when AMD develops [13]. They also reported that CC alteration precedes RPE loss and conclude that AMD can be classified as a vascular disease [13]. Examining 104 eyes of 80 patients suffering from AMD using OCTA, Spaide described that significant alterations were detectable in the flow pattern [36]. In a cohort of 42 eyes with intermediate AMD, Borrelli et al. later found that intermediate AMD eyes of patients with nAMD in the fellow eye have an increased average choriocapillaris signal void size compared to eyes without nAMD in the fellow eye [16]. In our study, the CC flow signal was significantly reduced after the application of Faricimab (*p* = 0.026, Table 2, Figure 1). The pathology of nAMD itself may be a possible reason for this finding. Another explanation is the effect of anti-VEGF and possibly the additional blockage of Ang2. Peters et al. reported that a significant loss of CC endothelial cell fenestrations was detected after intravitreal injection of Bevacizumab in primate eyes [20]. In another study, absence of soluble VEGF isoforms in mice led to age-dependent degenerative changes in the RPE-CC complex that recapitulates the classical features of dry AMD [35]. Falling in line with our observation, Hikichi et al. examined the vessel density of the CC in 124 nAMD eyes receiving anti-VEGF (Ranibizumab and Aflibercept) and found a significant decrease in CC vessel density over a follow-up period of 14.8 ± 3.1 months [21]. During this time, patients had received 3.6 ± 3.0 injections. The fact that we found a similar impact in CC flow signal after fewer injections (3.3 ± 1.6 vs. 3.6 ± 3.0) and in a shorter time frame (14.8 ± 3.1 months vs. 11 months) could indicate a stronger impact of Faricimab compared to Ranibizumab and Aflibercept.

Ang2 is part of the Ang/Tie pathway and is involved in the regulation of vascular homeostasis, the modulation of vascular permeability and in neoangiogenic and proinflammatory processes [26]. The pro- or anti-angiogenic activity of Ang2 is context dependent. One of the parameters that define Ang2 activity is the expression of other angiogenic growth factors, such as VEGF [37]. It was reported that in vitro, Ang2 induced permeability and angiogenesis on the pupillary membrane in the presence of VEGF, but in the absence of VEGF, Ang2 led to vessel regression and endothelial cell death [38]. Even though great care must be taken to transfer these findings to a clinical scenario, it is imaginable that the blockage of Ang2, especially together with depletion of VEGF could influence the permeability of choroidal vessels.

Previous studies reported that choroidal thickness in nAMD eyes is lower than in non-neovascular AMD eyes, indicating that not only is the CC affected, but the rest of the choroid is affected too [32]. Razavi et al. describe a similar finding and speculate that in nAMD, anti-VEGF treatment may favorably influence not only the retinal exudation, but also the underlying choroidal exudation by reducing choroidal vascular hyperpermeability [39].

Data about the behavior of HL and SL layers in the setting of AMD are controversial. In a study including 3187 participants, Zhao et al. found that the thickness of HL and SL was enlarged in AMD eyes compared to normal eyes [40]. In another study, the thickness was significantly reduced in the setting of AMD [41]. In our cohort, the flow signal of HL remained stable, whereas the SL flow signal increased. This could possibly be explained by a redirection of blood flow. Also, strong variations in choroidal thickness and flow signal following a diurnal rhythm have been reported, making this a limitation of our study [42].

In our study, CRT decreased significantly after one (T2) and at least two injections (T3) when compared to the baseline. This is coherent with the findings of the TENAYA and LUCERNE trials, even though the patients in our study were not treatment-naïve [43,44]. In contrast to the results of the TENAYA and LUCERNE trials, we did not find a significant change in BCVA. This is most likely caused by the advanced state of nAMD in our cohort. For the present analysis, we included only patients who had previously been treated with other anti-VEGF agents before administering Faricimab. Many of the patients had already been treated for multiple years (up to 10 years), so morphological changes were already advanced. This is a notable limitation of our study, because it may also have an influence on choroidal health.

To our current knowledge, this is the first study investigating the impact of intravitreally administered Faricimab on choroidal flow signal. The retrospective nature and the small sample size are notable limitations. Also, all of our patients had received previous treatment with other anti-VEGF agents. The goal of this current project was to specifically investigate the effect of switching from other anti-VEGF agents to Faricimab due to poor treatment response. All included patients had received extensive anti-VEGF treatment beforehand (15.9 ± 15.3 injections) and then received Faricimab treatment due to persistent disease activity. Investigations in this field are of high interest, as the exact mechanism leading to persistent disease activity despite anti-VEGF treatment is still not fully understood [45]. Moreover, many practitioners still do not use Faricimab as a first-line therapy, but as an option for patients with poor response to other established anti-VEGF drugs, making studies in this setting even more valuable. Faricimab counters not only VEGF, but also Ang2, making it a potent candidate for poor responders to solely anti-VEGF treatment [25]. Including patients who had previously received extensive anti-VEGF treatment could provide new data of the effect of blocking the Ang2 pathway by the drug, as this cohort had shown to be at least partly resistant to anti-VEGF.

A strength of the study lies in the utilization of a swept-source OCTA device, allowing to produce high-quality images. OCTA analysis of the deeper choroidal layers (including HL and SL) is controversial. In some cases, both eyes of the same individual were included in the study. In these cases, a correlation between the eyes cannot be fully excluded. The main challenges appear to be projection artefacts, limited signal penetration and resulting image resolution [46]. Using a swept-source OCTA device and a strict selection of only high-quality images, we addressed this issue [46,47]. Also, we applied the integrated signal compensation algorithm, an automatic and manual segmentation error correction and used validated local threshold strategies to maximize reliability. For this study we chose to analyze whole en face images, including the MNV. For this reason, changes in MNV morphology could have impacted the pixel count. However, the fact that all patients had received extensive anti-VEGF treatment beforehand (15.9 ± 15.3 injections) suggests that the MNV membrane was already in a steady state regarding its morphology at the time of the analysis. Manual segmentation of the MNV enabling exclusion of its flow signal as well as concentrating on the flow signal adjacent to the MNV have before been shown useful in the literature and could therefore be applied in future studies [24].

## 5. Conclusions

In conclusion, our study reports a decrease in CC flow signal after the administration of a series of at least 2 Faricimab injections. The flow signal of HL and SL remained stable. Furthermore, these results need to be interpreted with caution until further studies are conducted with treatment-naïve patients. In future, larger prospective studies are needed to support our preliminary results.

## Figures and Tables

**Table 1:** The table gives an overview over the intravitreal injections that patients had received before the treatment was changed to Faricimab. Numbers are presented as mean ± standard deviation.

	Mean (±SD)
injections per eye	15.9 ± 15.3
Ranibizumab	13.8 ± 12.8
Aflibercept	5.1 ± 3.4
Brolucizumab	10 ± 0
interval between injections (weeks)	5.4 ± 1.1

**Table 2:** The table gives an overview of the study cohort. Age is presented as mean ± standard deviation.

Age (Years)	78.4 ± 6.5
	N (%)
included eyes	29 (100)
included patients	25 (100)
male/female	6 (24)/19 (76)
right/left	12 (51)/17 (59)
≥2 Faricimab injections	4 (14)
≥3 Faricimab injections	25 (86)

**Table 3:** The table shows flow signal and central retinal thickness (CRT) measurements at different time points. CC = choriocapillaris, HL = Haller’s layer, SL = Sattler’s layer; data are presented as mean ± standard deviation. * statistically significant (*p*-value < 0.05).

	T1	T2	T3
CC flow signal (%)	54.4 ± 4.0	52.9 ± 5.9	51.97 ± 6.9 *
HL flow signal (%)	54.1 ± 4.8	55.2 ± 4.8	55.8 ± 5.1
SL flow signal (%)	54.7 ± 3.5	55.0 ± 4.8	55.9 ± 4.4
CRT (µm)	352 ± 82	292 ± 54 *	301 ± 78 *

**Table 4:** The table shows the results of the statistical analysis. Significant factors are marked as bold. CRT = Central Retinal Thickness. CC = Choriocapillaris flow signal. R^2^: Variance fraction explained by significant factors. *p* = *p* value.

	CRT		CC	
	Global R^2^ = 0.12		Global R^2^ = 0.04	
	Coeff.	*p*	Coeff.	*p*
Age	1.8	=0.39	0.06	=0.65
Sex	20	=0.53	0.99	=0.64
Time				
T2 vs. T1	**−60.5**	**=0.001**	−1.5	=0.25
T3 vs. T1	**−52.7**	**=0.005**	**−2.7**	**=0.050**
Cons. Term	350.3	≤0.001	54.2	≤0.001

## Data Availability

The original contributions presented in this study are included in the article. Further inquiries can be directed to the corresponding author.
